# Psychometric Evaluation of the Chinese Version of a Weight-Related Eating Questionnaire Using an Item Response Theory Approach

**DOI:** 10.3390/nu14081627

**Published:** 2022-04-13

**Authors:** Mandy Ho, Robert Smith, Pui-Hing Chau, Cheuk-Yan Chung, Susan M. Schembre, Daniel Y. T. Fong

**Affiliations:** 1 School of Nursing, The University of Hong Kong, Hong Kong 999077, China; robert.d.smith.37@gmail.com (R.S.); phchau@graduate.hku.hk (P.-H.C.); cychunggrace@gmail.com (C.-Y.C.); dytfong@hku.hk (D.Y.T.F.); 2 Department of Oncology, Georgetown Lombardi Comprehensive Cancer Center, Georgetown University, Washington, DC 20007, USA; ss4731@georgetown.edu

**Keywords:** eating behavior, emotional eating, dietary restraint, body mass index, psychometrics, questionnaire and survey

## Abstract

Valid and reliable measures are needed to identify individuals at risk of dietary restraint, emotional and external eating, and to customize weight loss education for more effective weight management. This study aimed to develop and validate a Chinese version of the Weight-Related Eating Behavior Questionnaire (WREQ-C) for assessing dietary restraint, emotional eating, and external eating. In stage one, the linguistic validation of the original English version of the WREQ (WREQ-E) was conducted. In stage two, the psychometric properties of the WREQ-C were first evaluated by item response theory-based (IRT) analyses. The reduced scale was then examined for convergent validity, structural validity (using a confirmatory factor analysis), population invariance, and test–retest reliability. The study included 1007 adults aged between 18 and 71 years. The IRT analysis optimally shortened the original WREQ-E from 16 to 13 items. A convergent validity analysis showed significant correlations between the WREQ-C subscales and the Chinese version of the Dutch Eating Behavior Questionnaire subscales (*r* = 0.63–0.82). The 13-item WREQ-C demonstrated good reliability (Cronbach’s α = 0.74–0.89) and validity for assessing the psychological aspects of eating behavior, including routine restraint, compensatory restraint, susceptibility to external cues, and emotional eating in Chinese adults.

## 1. Introduction

Obesity is a global public health problem with many detrimental effects on both physical and psychological health [1]. Dietary intake is a key component of obesity prevention and treatment. A meta-analysis of 34 epidemiological and observational studies reported that more than 40% of the world’s adult population is trying to lose weight [2]. However, the prevalence of obesity remains high. Most previous studies regarding obesity have focused on identifying and educating individuals on what and how much to eat. However, eating episodes and food choices are not solely controlled by the physiological demands for food. In an obesogenic environment, individuals are exposed to a plethora of personal, social, and environmental food-related cues, which encourage the consumption of energy-dense foods. Theory-based psychological aspects of eating behavior include dietary restraint (or restrained eating), external eating, and emotional eating. They are posited to be associated with overeating and high energy intake, and thus contribute to obesity and weight regain after successful weight loss [3,4]. Understanding the psychological aspects of eating behaviors is a critical area in obesity research. 

China is in the midst of an alarming increase in obesity [5]. In 2015, China had the second highest number of obese adults in the world, following the United States [6]. The influence of the psychological aspects of eating behavior on a person’s food choices and weight status in Chinese populations is an under-researched area. Valid and reliable measures are needed to identify individuals at risk of dietary restraint, emotional eating, and external eating, to tailor prevention and treatment strategies for more effective weight management, and to evaluate the effect of behavioral interventions.

Several questionnaires are available to assess the psychological aspects of eating behaviors. The most widely used tools are the Three-Factor Eating Questionnaire (TFEQ) and the Dutch Eating Behavior Questionnaire (DEBQ). The TFEQ is a 51-item questionnaire that includes three subscales: restraint, disinhibition, and hunger [7]. Despite the widespread use of the TFEQ in eating behavior research, its construct validity, factor structure, and factor stability have been criticized [8,9,10]. For example, the TFEQ does not have separate scales for emotional and external eating, which are nested within the disinhibition and hunger scales. The DEBQ is a 33-item, self-administered questionnaire that assesses external, emotional, and restrained eating behaviors [11]. Both the TFEQ and DEBQ are limited by a subject burden as a result of questionnaire length.

The Weight-Related Eating Behavior Questionnaire (WREQ) is a shorter, 16-item instrument that combines the strengths of the TFEQ and DEBQ and incorporates items reflecting new knowledge regarding eating behaviors [12]. Similar to the DEBQ, the WREQ assesses external and emotional eating. However, the WREQ captures the additional construct of dietary restraint and allows for the assessment of routine and compensatory restraints. Routine restraint reflects the perceived routine restriction of energy intake to control weight (a rigid restrictive eating pattern). Compensatory restraint reflects a more flexible approach to weight control, characterized by the intentional restriction of energy intake before and after an episode of perceived overeating. The psychometric properties of the original English version of the WREQ (WREQ-E) have been examined in various populations, including under/normal weight and overweight/obese adults [12,13]. The WREQ-E demonstrated a strong convergent validity (*r* = 0.736 to 0.849, *p* < 0.01) against the DEBQ and TFEQ, and food criterion-related validity against measures of dietary intake (dietary fat, and fruit and vegetable intake), in a sample of 482 college students in the US [12]. In addition, it has been tested in a multi-ethnic sample (including white, Asian/Asian-mix, and Native Hawaiian/Pacific Islanders) of adults (aged between 18 and 81 years) and demonstrated excellent criterion-related validity with respect to current weight status, weight change over time periods and weight control status, and an excellent test–retest reliability with interclass correlations of 0.849–0.932 [13].

Currently available tools for assessing the psychological aspects of eating behaviors are limited by a subject burden, as a result of questionnaire length. The item response theory (IRT) approach has been increasingly used to produce precise, valid, and relatively brief health instruments to reduce the response burden [14]. This study is the first to describe the transcultural adaptation and psychometric evaluation of the Chinese version of the WREQ (WREQ-C), to assess the underlying psychological aspects of eating behavior related to overeating in Chinese adults, using both the IRT and classical test theory approaches.

## 2. Materials and Methods

This was a questionnaire development and psychometric validation study and involved transcultural adaptation and psychometric testing of a weight-related eating behavior questionnaire with a two-stage design. The Institutional Review Board of the University of Hong Kong/Hong Kong West Cluster, the Hospital Authority in Hong Kong, approved the research protocol (approval number: UW19-122). Participants signed an online consent form.

### 2.1. Weight-Related Eating Questionnaire

The original English version of the WREQ (WREQ-E) [12] is a 16-item questionnaire that consists of four subscales: routine restraint (three items), compensatory restraint (three items), susceptibility to external cues (five items), and emotional eating (five items). All items were measured using a five-point Likert scale (1—not at all, 2—sometimes, 3—half of the time, 4—most of the time, 5—always). The routine restraint construct assesses the perceived routine restriction of energy intake to control weight. The compensatory restraint construct assesses the intentional restriction of energy intake following an episode of overeating. The susceptibility to external cues construct assesses eating in response to external cues without regard for internal signals of hunger or satiety. The emotional eating construct assesses eating in response to negative emotions.

### 2.2. Stage 1: Translation and Transcultural Adaptation of WREQ

Translation and transcultural adaptation was conducted according to the World Health Organization guidelines for the process of translation and adaptation [15]. Firstly, the WREQ-E was translated from English to Chinese by two health professionals who are native Chinese speakers, have good English language skills, and are familiar with eating behavior terminology. The translated version of WREQ-C was then back-translated into English by two independent bilingual translators, who are native speakers of English but are also proficient in Chinese and have no prior knowledge of the WREQ-E. Culture strongly influences people’s eating behavior. Therefore, an expert panel including a doctor, nurse, psychologist, public health nutritionist, and two dietitians who work in the field of obesity were invited to independently review the content representativeness and cultural relevance of the items in the translated version, WREQ-C, and to suggest new questions deemed appropriate for Chinese populations. The representativeness and relevance were assessed using a five-point Likert scale (1—not at all, 2—slightly, 3—more or less, 4—pretty well, 5—completely). A content validity index (CVI) was calculated by identifying the proportion of experts who perceived the representativeness/relevance of the question items as a 4 or 5 on the scale. First, the item-level content validity index (I-CVI) was calculated for each item. Items that scored >0.8 were retained. In this study, all items were retained. The scale-level content validity index (S-CVI) was then determined from the average of the I-CVI scores for all items on the scale. The S-CVI of representativeness and relevance were 0.97 and 0.98, respectively, which are considered to be good content validity [11]. Six new question items (three items each for the emotional and susceptibility to external cues subscales) were added to the WREQ-C according to the panel’s comments, resulting in a 22-item version of the questionnaire (Supplementary Materials Table S1).

#### Pilot Test

The expert panel-modified version of the 22-item WREQ-C was trialed on 18 Chinese adults. Participants were asked to complete an online questionnaire and attend a debriefing interview (either face-to-face or via telephone) to provide comments regarding the relevance and clarity of the questionnaire items.

### 2.3. Stage 2: Psychometric Evaluation of WREQ-C

#### 2.3.1. Sample Size Calculation

As the WREQ-E includes 16 items, the WREQ-C was expected to be of similar length. A sample size of at least 160 participants was calculated for this study based on a subject-to-item ratio of 10:1 [16]. To accommodate for the possibility of developing additional culture-specific items and to ensure a power of 80% to detect a significant difference between underweight, normal weight, overweight, and obese groups at the two-tailed level of 5%, at least 288 participants were required (using local, population-based survey data regarding weight status).

#### 2.3.2. Data Collection

Data were collected in the form of an online survey, consisting of the 22-item WREQ-C, the 33-item DEBQ, and demographic questions, including self-reported weight, height, and weight control status. The request response option was used in the online survey to increase the question response rate. If a respondent skipped/missed a question, the online platform would remind respondents that they missed a question and ask if they would like to go back and answer the skipped/missed question before they move to a new page.

#### 2.3.3. Participant Recruitment

Participants were recruited from the community via convenience sampling using various strategies, including distributing recruitment materials to community groups, via university mass emails and other social media (such as Facebook and websites) to complete an online survey. Chinese adults aged 18 years or older, irrespective of their body weight (underweight, normal weight, overweight, or obese) and weight control status, who were able to read Chinese and speak Cantonese, were included in the study. Individuals with a health condition that required dietary restrictions (such as diabetes, renal failure, or food allergies) or a history of or a current eating disorder diagnosis (such as anorexia nervosa or bulimia nervosa), and those who were pregnant or lactating during the study period, were excluded. The final sample included 1007 participants, which were randomly divided into questionnaire development and validation samples. The development sample (*n* = 504) was used to refine the structure of the WREQ-C, including the evaluation of new items in the IRT models. The validation sample (*n* = 503) was used to determine the structural validity, measurement invariance, and convergent validity of the WREQ-C.

#### 2.3.4. Development of the New WREQ-C

Data from the development sample were used to evaluate the items within each subscale of the WREQ-C using polytomous generalized partial-credit item IRT models. Separate models were conducted for each subscale, and the uni-dimensionality was evaluated using exploratory factor analyses. The items and subscales were evaluated using three criteria (Box 1). In subscales where all these criteria were met, the item with the lowest discriminative value was removed until the new structure of the subscale failed in one of these criteria (item reduction). The reduced scale was then examined for content validity, convergent validity, structural validity, measurement invariance, and test–retest reliability.

Box 1 **Criteria for item and subscale evaluation in the development of the new Chinese version of the Weight-Related Eating Behavior Questionnaire (WREQ-C).**

(1) Test information of the subscale had to be maintained within 80% of the original structure of the 16-item English version of the Weight-Related Eating Behavior Questionnaire (WREQ-E)
(2) The subscales retained adequate convergent construct validity (*r* > 0.6) as compared to the relevant Dutch Eating Behavior Questionnaire (DEBQ) subscale
(3) The internal consistency of the subscales needed to achieve a Cronbach’s α ≥ 0.7

#### 2.3.5. Content Validity

To confirm content validity, the finalized version of the WREQ-C was reviewed by an expert panel that included two dietitians, one registered nurse who works in the field of obesity, and an expert in instrument development and cultural adaptation of health measurements. Each item removed from the WREQ-C was thoroughly discussed to ensure that the content validity was not affected.

#### 2.3.6. Convergent Validity

The 33-item Chinese version of the DEBQ [17] was used as a reference for the convergent validity of the WREQ-C. The DEBQ is a well-established, self-administered questionnaire that measures restraint eating (10 items), susceptibility to external cues (10 items), and emotional eating (13 items), using a five-point Likert scale. 

#### 2.3.7. Structural Validity

The structural validity of the WREQ-C was assessed using confirmatory factor analysis (CFA). A four-correlated factor model (routine restraint, compensatory restraint, susceptibility to external cues, and emotional eating) was fitted using weighted least squares mean, and variance-adjusted estimators for categorical variables was used to model the observed polychoric correlation matrix. Model fit statistics to evaluate goodness-of-fit indictors included chi-square, comparative fit index (CFI), Tucker–Lewis index (TLI), weighted root mean square residual (WRMR), and root mean square error of approximation (RMSEA). Acceptable model fit was set as CFI ≥ 0.95 [18], TLI ≥ 0.95, RMSEA ≤ 0.06 [19], and WRMR ≤ 1.00 [20].

#### 2.3.8. Measurement Invariance

A factorial invariance analysis was performed using a set of CFA models to identify any bias within the structural validity of the WREQ-C between subgroups based on weight statuses (underweight or normal weight vs. overweight or obese), age (≤29 years vs. ≥30 years), and sex (male vs. female). Measurement invariance was evaluated using a configural (unconstrained) model that progressed to an increasingly constrained model. The models were constrained by factor loading, where factor loadings were constrained to be equal between groups, and item intercepts where item thresholds were constrained to be equal between groups. The residual variances of the items were fixed at one in the first group (as listed above) and freely estimated in the comparator group. Measurement invariance was evaluated using the change (Δ) in degrees of freedom (df) between the configural and constrained models. The change in CFI (ΔCFI) (CFI_contstrained model_−CFI_configural model_) was also used to measure invariance between the subgroups. A ΔCFI ≤ 0.01 was considered the best index for indicating invariance within the models for each subgroup [21].

#### 2.3.9. Test–Retest Reliability

A separate sample of 31 participants with different weight statuses and education levels completed the questionnaire for a second time after a two-week interval. The test–retest reliability was assessed using two-way random effects intra-class correlations (ICC) for absolute agreement for each subscale of the WREQ-C. An ICC > 0.7 represented adequate reliability [22]. 

### 2.4. Statistical Analysis

Continuous variables are presented as means and standard deviations, and categorical variables are presented as frequencies and proportions. Most analyses were conducted using Stata Statistical Software Release 16 (StataCorp, College Station, TX, USA). The MIRT package for R (R Core Team, R Foundation, Austria, Vienna) [23] was used to conduct the IRT analysis. The CFA and measurement invariance analysis were conducted using Mplus version 7.4 (Muthén and Muthén, Los Angeles, CA, USA) [24]. Due to the online platform data were collected from, there were no missing data in returned questionnaires among the 1007 participants.

## 3. Results

A total of 1085 questionnaires were received, and 78 respondents (7%) did not complete any of the WREQ items and were excluded from analyses, resulting in a sample of 1007. There were no significant differences in demographic characteristics between the completers (*n* = 1007) and non-completers (*n* = 78). Of the 1007 participants in this study, 739 (73%) were female. The mean body mass index (BMI) was 21.6 ± 3.7 kg/m^2^. The participants’ characteristics are shown in Table 1. The participants’ ages ranged from 18 to 71 years, and most participants (68%) were single, employed full-time (48%), and had a bachelor’s degree or above (78%). There were no significant differences in demographic characteristics between the development (*n* = 504) and validation (*n* = 503) samples (Table 1). 

### 3.1. Development of the WREQ-C

#### 3.1.1. Routine Restraint Subscale

**Figure 1:**
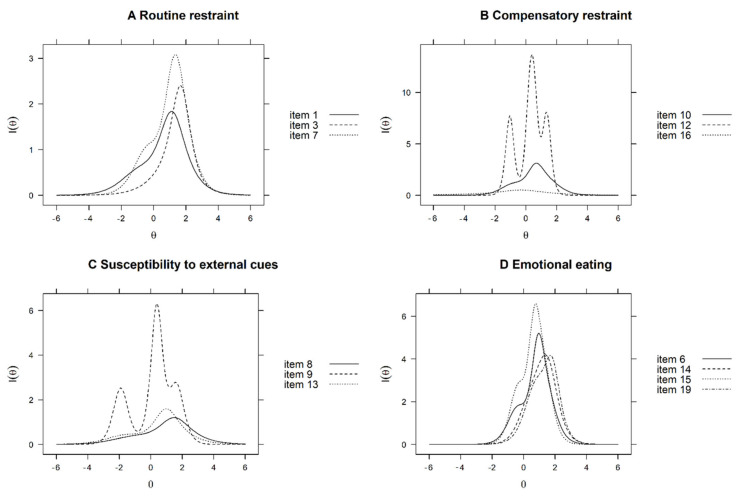
Item information function for the new structure to domains of the WREQ-C. (**A**) Routine restraint, (**B**) compensatory restraint, (**C**) susceptibility to external cues, and (**D**) emotional eating. Latent trait (Theta(θ)) is shown on the horizontal axis, and the amount of information (I(θ)) is shown on the vertical axis. WREQ-C, Chinese version of the Weight-Related Eating Behavior Questionnaire.

The three-item structure of the original WREQ-E was retained for the routine restraint subscale. The test information was 17.5, and the routine restraint maintained a correlation with the DEBQ-restrained eating subscale (*r* = 0.71, *p* < 0.001), with an internal consistency of 0.76. Item 3 had the lowest discriminative value, but Cronbach’s alpha dropped below the criteria threshold when it was removed (0.66). Therefore, this item was retained in the final version of the WREQ-C. The test information curves regarding routine restraint displayed items providing more information at higher levels (θ = 0) of the latent trait (Figure 1A).

#### 3.1.2. Compensatory Restraint Subscale

The three-item structure for compensatory restraint was maintained. The test information was 32.5 and correlated to the DEBQ-restrained eating subscale (*r* = 0.61, *p* < 0.001), with a Cronbach’s alpha of 0.78. The test information decreased to less than 80% of that of the original WREQ-E when item 16 was removed (the test information of the 2-item structure was 22.4, which was 69% of the WREQ-E). Therefore, item 6 was retained in the final version of the WREQ-C. Test information curves regarding compensatory restraint items present more information at three separate levels of the latent trait (Figure 1B).

#### 3.1.3. Susceptibility to External Cues Subscale

The original five-item structure of the susceptibility to external cues subscale (items 5, 8, 9, 11, and 13) had a test information area of 23.4, and three of the newly developed items were included in the susceptibility to external cues subscale in the new WREQ-C (items 20, 21, and 22). After adding the new items, the item with the lowest discriminative value was removed until the criteria (Box 1) could not be maintained, resulting in a three-item structure (including items 8, 9, and 13). The test information area of the new three-item structure was 21.7 (which was 93% of the original WREQ-E). Item 5 (“I tend to eat more food than usual when I have more available places near home, workplace, or study place that serve or sell food”) and item 11 (“I often eat so quickly I don’t notice I’m full until I’ve eaten too much”) were removed. Removing any further items resulted in a test information area of less than 80% of the original structure. Each excluded item was reviewed by an expert panel to ensure that the content validity was not affected by their removal. The three-item structure of susceptibility to external cues correlated with the susceptibility to external cues subscale of the DEBQ (*r* = 0.62, *p* < 0.001). The Cronbach’s alpha of the new structure remained above the threshold criterion (0.72). Test information curves for the new structure displayed items providing more information at a higher ability of the average level (θ = 0) of the latent trait (Figure 1C).

#### 3.1.4. Emotional Eating Subscale

The original five-item structure of the emotional eating subscale (items 2, 4, 6, 14, and 15) had a test information of 48.5. Three of the newly developed items were added to the emotional eating subscale (items 17, 18, and 19). After item reduction, a four-item structure including items 6, 14, 15, and 19 was developed. The test information of the new four-item structure was 40.5 (83% of the original structure). Item 2 (“I tend to eat more when I am anxious, worried, or tense”) and item 4 (“When I feel lonely, I console myself by eating”) were removed. Removing any further items resulted in a test information area of less than 80% of the original structure. The new four-item structure of emotional eating correlated with diffuse emotion (*r* = 0.73, *p* < 0.001), labeled emotion (*r* = 0.79, *p* < 0.001), and emotional eating (*r* = 0.81, *p* < 0.001) of the DEBQ. Cronbach’s alpha for the new structure remained above the threshold criterion (0.89). Test information curves for the new structure of emotional eating items present more information at a higher ability of the average level (θ = 0) of the latent trait (Figure 1D). The IRT analysis resulted in a 13-item WREQ-C structure.

#### 3.1.5. Structural Validity

**Figure 2:**
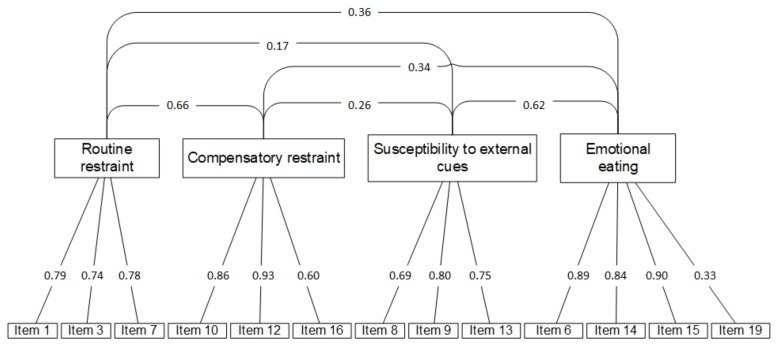
Correlated confirmatory 4-factor analysis model of the 13-item WREQ-C (*n* = 1007).

The CFA of the 13-item WREQ-C resulted in a 4-factor model with an acceptable fit to the validation data using the validation sample. The correlations between items and their designated factors were strong (standardized β > 0.6), with the exception of item 19 (Figure 2). Goodness-of-fit indicators showed acceptable fit in CFI = 0.97, TLI = 0.97, and RMSEA = 0.06; however, the WRMR (WRMR = 1.22) did not meet the predetermined model fit criteria and the chi-square value was 277.45 (df = 59, *p* < 0.001). 

#### 3.1.6. Measurement Invariance

Table 2 shows the models that were acceptable for CFI (>0.90), but not for RMSEA or WRMR in males and females (chi-square value = 777.18, df = 260, *p* < 0.001; CFI = 0.96; RMSEA = 0.07; WRMR = 1.98), adults aged ≤ 29 years and those aged ≥ 30 years (chi-square value = 932.12, df = 260, *p* < 0.001; CFI = 0.95; RMSEA = 0.76; WRMR = 2.04), and underweight or normal weight and overweight or obese (chi-square value = 939.57, df = 260, *p* < 0.001; CFI = 0.96; RMSEA = 0.08; WRMR = 2.08). The comparison between the two constrained models suggested invariance in factor loading and item intercepts between genders (Δ chi-square value = 14.68, Δdf = 8, *p* < 0.001; ΔCFI = 0.01; ΔRMSEA = 0.01), BMI status (Δ chi-square value = 20.01, Δdf = 8, *p* < 0.001; ΔCFI = 0.01; ΔRMSEA = 0.01), and age groups (Δ chi-square value = 20.10, Δdf = 8, *p* < 0.001; ΔCFI = 0.01; ΔRMSEA = 0.01) (Table 2). This means that the factor structure, factor loadings, and indicator intercepts were consistent between males and females, and across various weight statuses and age groups.

#### 3.1.7. Test–Retest Reliability

The test–retest subsample included 31 adults (19 females, 61%) aged between 18 and 65 years with a mean BMI of 23.4 ± 4.9 kg/m^2^. The mean number of days between the first and second administrations of the WREQ-C was 17.6 ± 4.9. The ICC for all subscales was high (routine restraint ICC = 0.76, 95% CI: 0.55–0.87, *p* < 0.001; compensatory restraint ICC = 0.76, 95% CI: 0.55–0.87, *p* < 0.001; susceptibility for external cues ICC = 0.78, 95% CI: 0.57–0.91, *p* < 0.001; emotional eating ICC = 0.89, 95% CI: 0.77–0.97, *p* < 0.001).

## 4. Discussion

In this study, a novel WREQ-C was developed and validated based on the WREQ-E and DEBQ. This is the first study to extensively test the psychometric properties and measurement invariance of a Chinese version of the eating behavior instrument for assessing dietary restraint, susceptibility to external cues, and emotional eating in adult populations. The original WREQ-E was modified from 16 to 13 items with little information lost, while maintaining validity and reliability [12]. The resulting 4-factor, 13-item WREQ-C demonstrated satisfactory convergent validity with the corresponding subscales of the DEBQ, good internal consistency, and good test–retest reliability. The structural validity was generally good, and the results of the measurement invariance tests indicated equivalent factor loadings and item intercepts between subgroups of different sexes, BMIs, and ages. These results suggest that the shortened version of the WREQ-C is a valid and reliable instrument for the adult population with various weight statuses and age groups.

Culture has a strong influence on an individual’s eating behavior, and six new question items (items 17–22) were proposed by the expert panels. The IRT-based analyses of these new items showed that only one item (item 19: “I eat more when I am having relational problems with my family”) provided additional information, and thus was retained in the WREQ-C. This reflects the role that family relationships play in emotional eating among Chinese adults. Additionally, the findings of this study demonstrate the robustness of the original WREQ-E for the assessment of eating behaviors across various cultures. 

Obesity is a major public health problem and is increasing in China. As the psychological aspects of eating behavior, including susceptibility to external cues, emotional eating, and restraint eating, are posited to be associated with overeating and high energy intake and to contribute to obesity, a reliable and valid instrument to assess these specific eating behavior tendencies is critical in both obesity research and clinical practice. Firstly, it can be used as an assessment tool for healthcare providers to identify individuals at risk of overeating, and to tailor education for more effective weight management, thereby preventing or treating obesity and related complications. Secondly, WREQ can be used as an evaluation tool for the effects of behavioral interventions on the psychological aspects of eating behavior. Finally, weight regain is a common phenomenon for weight loss intervention. Long-term behavioral self-regulation is an important factor of long-term successful weight control. WREQ will be a useful assessment tool for understanding the psychological factors that may contribute to the long-term weight loss and weight loss maintenance. Understanding the psychological factors contributing to obesity, weight loss, and weight maintenance may enable policy makers, clinicians, and researchers to develop and implement appropriate educational and environmental strategies to achieve positive outcomes.

Compared with the existing tools, such as TFEQ and DEBQ for assessing psychological eating behaviors, WREQ is unique in that it allows for the assessment of two subscales of dietary restraint, namely the routine restraint and compensatory restraint, respectively. The routine restraint subscale assesses the perceived routine restriction of energy intake to control weight, which may reflect rigid control of energy intake, whereas the compensatory restraint assesses the intentional restriction of energy intake following an episode of overeating, reflecting a more flexible control of energy intake. Research shows that flexible and rigid controls of dietary intake have differential relationships to disordered eating and BMI [25]. Flexible control of dietary intake was negatively associated with BMI, whereas rigid control was positively associated with BMI [26,27,28]. 

Another strength of the modified version of the WREQ-C is that it is a shorter instrument (13 items) than similar instruments measuring the susceptibility to external cues, emotional eating, and restraint eating, including the DEBQ (33 items) and TFBQ (51 items). Therefore, this may reduce the response burden and increase completion rates, which is beneficial for both researchers and clinicians.

The major limitation of this study is that the study samples were recruited by convenience sampling, which may limit the generalizability of the findings to other populations. However, the large sample size of this study and successful recruitment of participants with various body weight statuses and ages allowed for a comprehensive evaluation of the psychometric properties of the WREQ-C. In addition, the IRT used in this study was sample-independent. This study was also limited by the use of self-reported body weight and height to estimate weight status. Therefore, applying the WREQ-C to diverse populations and collecting measured weight data is recommended for future studies. In addition, the data of this validation study were collected using an online questionnaire. Further studies may be required to validate the printed version of WREQ.

## 5. Conclusions

The novel 13-item WREQ-C demonstrated good reliability and validity for assessing the psychological aspects of eating behavior, including routine restraint, compensatory restraint, susceptibility to external cues, and emotional eating, in Chinese adults. It can be used to identify the underlying psychological aspects of eating behaviors related to overeating and obesity, and to design more precise and effective educational and environmental strategies to reduce the obesity epidemic.

## Figures and Tables

**Table 1:** Descriptive statistics of the study sample (*n* = 1007).

	Total (*n* = 1007)	Development Sample (*n* = 503)	Validation Sample (*n* = 504)	*p*-Value ^1^
Gender	*n* = 1006	*n* = 503	*n* = 504	0.872
Females	739 (73)	368 (73)	371 (74)	
Mean age in years (SD)	32.6 (13.7)	32.5 (13.5)	32.7 (13.9)	0.874
Age groups	*n* = 1007	*n* = 503	*n* = 504	0.468
Aged 29 years or younger	562 (56)	275 (55)	287 (57)	
Aged 30 years or above	445 (44)	228 (45)	217 (43)	
Mean BMI kg/m^2^ (SD)	21.3 (3.0)	21.8 (3.7)	21.5 (3.6)	0.367
BMI category	*n* = 949	*n* = 471	*n* = 478	0.787
Underweight	139 (15)	68 (14)	71 (15)	
Normal weight	508 (54)	244 (52)	264 (55)	
Overweight	136 (14)	71 (15)	65 (14)	
Obese	166 (17)	88 (19)	78 (16)	
Self-reported Health status	*n* = 1007	*n* = 503	*n* = 504	0.783
Extremely well	24 (2)	13 (3)	11 (2)	
Very well	240 (24)	128 (25)	112 (22)	
Well	361 (36)	177 (35)	184 (37)	
Fair	351 (35)	170 (34)	181 (36)	
Bad	31 (3)	15 (3)	16 (3)	
Marital status	*n* = 1006	*n* = 502	*n* = 504	0.037
Single	684 (68)	344 (69)	340 (68)	
Married	294 (29)	147 (29)	147 (29)	
Divorced/Separate/widowed	28 (3)	11 (2)	17 (3)	
Employment status	*n* = 1007	*n* = 503	*n* = 504	0.298
Full-time	481 (48)	258 (51)	223 (44)	
Part-time	37 (4)	16 (3)	21 (4)	
Retired/unemployed/homemaker	78 (8)	33 (7)	45 (9)	
Student	411 (41)	196 (39)	215 (43)	
Education level	*n* = 1006	*n* = 503	*n* = 503	0.097
Senior secondary or below	118 (12)	56 (11)	62 (12)	
Diploma/certificate/associate degree	104 (10)	57 (11)	47 (9)	
Bachelor’s degree	510 (51)	251 (50)	259 (52)	
Master’s degree or above	274 (27)	139 (28)	135 (27)	
Family monthly income (HKD)	*n* = 1000	*n* = 498	*n* = 502	0.724
<9999	110 (11)	53 (11)	57 (11)	
10,000–19,999	178 (18)	86 (17)	92 (18)	
20,000–29,999	196 (20)	98 (20)	98 (20)	
30,000–39,999	154 (15)	72 (15)	82 (16)	
40,000–59,999	170 (17)	95 (19)	75 (15)	
60,000 or above	192 (19)	94 (19)	98 (20)	
Mean WREQ-C score (SD)	*n* = 1007	*n* = 503	*n* = 504	
Routine restraint (1–5)	2.0 (0.9)	2.0 (0.8)	2.0 (0.9)	0.516
Compensatory restraint (1–5)	2.8 (1.0)	2.8 (1.0)	2.8 (1.0)	0.274
External eating (1–5)	2.5 (0.8)	2.5 (0.8)	2.6 (0.9)	0.262
Emotional Eating (1–5)	2.1 (0.9)	2.0 (0.9)	2.0 (1.0)	0.465

SD, standard deviation; BMI, body mass index; HKD, Hong Kong Dollar; WREQ-C, Chinese version of the Weight-Related Eating Behavior Questionnaire. Values are number of participants (%) unless otherwise indicated. ^1^ *p*-values for comparison between the development and validation samples.

**Table 2:** Confirmatory factor analysis fit indices by age, gender, and body mass index (BMI).

							Factor Score Determinacy			
Subgroup	*n*	*X*^2^ (df)	CFI	TFI	RMSEA	WRMR	Compensatory Restraint	Routine Restraint	External Eating	Emotional Eating
Total	503	277.5 (59)	0.97	0.97	0.06	1.22	0.89	0.88	0.90	0.91
Males	128	215.6 (59)	0.95	0.94	0.09	1.07	0.88	0.87	0.91	0.91
Females	375	217.8 (59)	0.96	0.96	0.08	1.31	0.88	0.88	0.90	0.92
aged ≤ 29 years	255	205.4 (59)	0.95	0.95	0.09	1.19	0.87	0.88	0.90	0.90
aged ≥ 30 years	248	206.1 (59)	0.95	0.94	0.07	1.17	0.88	0.87	0.89	0.91
underweight/normal weight	326	210.5 (59)	0.96	0.96	0.08	1.26	0.91	0.90	0.92	0.92
overweight/obese	144	208.2 (59)	0.97	0.96	0.08	1.10	0.89	0.89	0.90	0.90

*X*^2^, chi-square; df, degrees of freedom; CFI, comparative fit index; TFI, Tucker–Lewis index; RMSEA, root mean square error of approximation; WRMR, weighted root mean square residual.

## Data Availability

The datasets used and/or analyzed during the current study are available from the corresponding author upon reasonable request.

## Supplementary Materials

The following supporting information can be downloaded at https://www.mdpi.com/article/10.3390/nu14081627/s1, Table S1: The 22-item Chinese version of the Weight-Related Eating Behavior Questionnaire (WREQ-C).
