# Supplementary material for: Psychometric Evaluation of the Chinese Version of a Weight-Related Eating Questionnaire Using an Item Response Theory Approach
Source: Nutrients. 2022 Apr 13;14(8):1627. doi: 10.3390/nu14081627 (PMC9031542; doi:10.3390/nu14081627)
Supplement: Supplementary file 1 [file nutrients-14-01627-s001.zip › nutrients-1645109-supplementary.pdf]

Table S1. The 22-item Chinese version of the Weight-Related Eating Behavior Questionnaire (WREQ-C).

1.	I purposefully hold back at meals in order not to gain weight.
^2.	I tend to eat more when I am anxious, worried or tense.
3.	I count calories as a conscious means of controlling my weight.
^4.	When I feel lonely, I console myself by eating.
^5.	I tend to eat more food than usual when I have more available places near home, workplace or study place that serve or sell food.
6.	I tend to eat when I am disappointed or feel let down.
7.	I often refuse foods or drinks offered because I am concerned about my weight.
8.	If I see others eating, I have a strong desire to eat too.
9.	Some foods taste so good I eat more even when I am no longer hungry.
10.	When I have eaten too much during the day, I will often eat less than usual the following day.
^11.	I often eat so quickly I don't notice I'm full until I've eaten too much.
12.	If I eat more than usual during a meal, I will try to make up for it at another meal.
13.	When I'm offered delicious food, it's hard to resist eating it even if I've just eaten.
14.	I eat more when I am having relationship problems.
15.	When I'm under a lot of stress, I eat more than I usually do.
16.	When I know I'll be eating a big meal during the day, I try to make up for it by eating less before or after the meal.
*^17.	I tend to eat more when I feel bored.
*^18.	Eating is the only way to improve my mood when I feel down.
*19.	I eat more when I am having relational problems with my family.
*^20.	When I smell a delicious food, it's hard to resist eating it even if I've just eaten.
*^21.	I tend to eat while I am preparing or cooking meals.
*^22.	I will finish everything on the plate even though I am already full.

Scoring protocol: Not at all = 1, Sometimes = 2, Half of the time = 3, Most of the time = 4, Always = 5; Routine Restraint = item 1, 3 and 7; Compensatory Restraint = item 10, 12 and 16; Susceptibility to external Cues = Item 5, 8, 9, 11, 13, 20, 21 and 22; Emotional Eating = item 2, 4, 6, 14, 15, 17, 18 and 19. *newly added question items; ^items removed in the final version of the 13-item WREQ-C. WREQ-C, Chinese version of the Weight-Related Eating Behavior Questionnaire.
